# FecB Was Associated with Litter Size and Follows Mendel’s Laws of Inheritance When It Transited to Next Generation in Suhu Meat Sheep Breeding Population

**DOI:** 10.3390/genes15030260

**Published:** 2024-02-20

**Authors:** Pengwei Su, Yifei Gu, Shanhe Wang, Xiukai Cao, Xiaoyang Lv, Tesfaye Getachew, Yutao Li, Zhenghai Song, Zehu Yuan, Wei Sun

**Affiliations:** 1 Joint International Research Laboratory of Agriculture and Agri-Product Safety of Ministry of Education of China, Yangzhou University, Yangzhou 225009, China; supengwei4901@163.com (P.S.); feilin133082@163.com (Y.G.); cxkai0909@163.com (X.C.); dx120170085@yzu.edu.cn (X.L.); yuanzehu@yzu.edu.cn (Z.Y.); 2 International Joint Research Laboratory in Universities of Jiangsu Province of China for Domestic Animal Germplasm Resources and Genetic Improvement, Yangzhou University, Yangzhou 225009, China; 007121@yzu.edu.cn; 3 College of Animal Science and Technology, Yangzhou University, Yangzhou 225009, China; 4 International Centre for Agricultural Research in the Dry Areas, Addis Ababa 999047, Ethiopia; t.getachew@cgiar.org; 5 CSIRO Agriculture and Food, 306 Carmody Rd, St Lucia, Brisbane, QLD 4067, Australia; yutao.li@csiro.au; 6 Dongshan Animal Epidemic Prevention Station of Wuzhong District, Suzhou 215000, China; 13451631997@163.com; 7 Innovative China “Belt and Road” International Agricultural Technology Innovation Institute for Evaluation, Protection, and Improvement on Sheep Genetic Resource, Yangzhou 225009, China

**Keywords:** FecB, Suhu meat sheep, inheritance patterns, litter size, early growth and development

## Abstract

In order to investigate the effect of FecB on litter size and growth and development traits of Suhu meat sheep and the inheritance patterns of FecB between parents and offspring in the population. In this experiment, 2241 sheep from the Suhu meat sheep population were tested for FecB using capillary electrophoresis. We combined the lambing records of 473 ewes, the growth trait records of 881 sheep at both the birth and weaning (2-month-old) stages, and the complete genealogical records of 643 lambs to analysis the distribution of FecB in the Suhu meat sheep breeding population, its effect on litter size of ewes, growth and development of lambs, and the inheritance patterns of FecB. The results showed that there were three genotypes of FecB in the Suhu meat sheep population, namely the AA genotype, AG genotype, and GG genotype. FecB in this population has a moderate polymorphism (0.25 < *PIC* < 0.5), and deviates from Hardy–Weinberg disequilibrium *(p* < 0.05). The litter size of GG genotype ewes was significantly higher than that with the AG and AA genotypes (*p* < 0.01). A Chi-square test showed that the inheritance patterns of FecB follows Mendel’s Laws of Inheritance (*p* > 0.05). An association analysis of different genotypes of FecB with body weight and body size of Suhu meat sheep at birth and weaning revealed that FecB adversely affects the early growth and development of Suhu meat sheep. In summary, FecB can improve the litter size of ewes but it has negative effects on the early growth and survival rate of lambs in sheep. Therefore, FecB test results and feeding management measures should be comprehensively applied to improve the reproductive performance of ewes, the survival rate and production performance of lambs in sheep production, and thus improve the economic benefits of sheep farms.

## 1. Introduction

FecB is a mutation site in which base A at position 746 of the bone morphogenetic protein receptor IB (BMPR-IB) gene on sheep chromosome 6 is mutated to G (A746G). It was firstly discovered in Booroola Merino sheep in 1989 and it could affect litter size in Booloola Merino sheep [1,2]. A missense mutation at this locus resulted in the conversion of glutamine to arginine (Q249R) [1,3], which altered the spatial structure of the BMPR-IB protein and then affect the expression of downstream genes to control follicle development [4]. Previous studies have shown that FecB can reduce the activity of the BMP signalling system during follicular development by inhibiting the BMP signalling pathway, leading to BMP inhibition of granular cells (GCs) proliferation and higher sensitivity to Follicle-stimulating hormone (FSH). This regulates the response of GCs and oocytes to Follicle-stimulating hormone (FSH) and Luteinising hormone (LH), leading to the accelerated maturation of follicles and ovulation at pre-estrus [5,6]. The main role of FecB is to increase the level of ovulation in ewes and then increase the litter size. FecB can increase the litter size in many sheep breeds, such as Small-tail Han sheep [7], Garole × Malpura sheep [8], Hu sheep [9], and Bayanbruk [10] sheep. However, FecB was not found in some sheep breeds, such as Dorper, Suffork, and Xinjiang sheep. This shows that FecB is breed specific. So, whether FecB is present in new sheep populations and what its role is in litter size still need further validation.

Currently, crossbreeding is an important method to breed a new sheep breed. Suhu meat sheep [11] is a breeding population that formed by our team. It is a crossbred population of Dorper and Hu sheep. Usually, specialized breeds, such as Dorper, which has an excellent performance in meat production, is selected as sires, and local sheep breeds with some qualified characteristics, such as Hu sheep, famous for its high prolificacy were selected as ewes. Unfortunately, the sires with high-yield meat sheep such as Dorper sheep usually do not carry the FecB mutation which could show a negative effect on litter size for its offspring. Selection for several generations is an essential process for breed a new sheep breed in a cross-breeding population. However, the genetic transmission pattern of FecB from parents to offspring is unclear which is useful for guiding mating.

Due to Hu sheep and its offspring mating with Dorper sheep, they carry FecB [12]. We hypothesise that FecB is correlated with litter size and early growth and conforms to a genetic transmission pattern, such as Mendel’s Laws of Inheritance from parents to offspring in Suhu meat sheep. In order to test this hypothesis, 2241 Suhu meat sheep with pedigree, lambing records, and early growth traits were selected as the research subjects. The genotype of FecB was detected by capillary electrophoresis. The association between FecB and litter size as well as early growth traits was analysed. The inheritance patterns of FecB was detected with pedigree information. Our research could provide a molecular marker for selection litter size in Suhu meat sheep and provide a theoretical basis for guiding mating.

## 2. Materials and Methods

### 2.1. Experimental Material

In this experiment, blood was collected from 2241 Suhu meat sheep in the period 2020–2023 and stored in a −20 °C refrigerator with sodium heparin anticoagulation tubes. Lambing records of 473 ewes, growth performance records of 881 lambs and pedigree records of 643 lambs were collected from Xuzhou Suyang Sheep Industry Co., Ltd. (Xuzhou, China). Among 473 ewes, 192 were primiparous ewes with about 1 year old, and 281 parturient ewes were 1.5–3.5 years old. Breeding was based on the genealogical records and they were unrelated within three generations. Theoretically, the value of the inbreed was less than 0.125. All the tested sheep were housed under the same feeding conditions, temperature, humidity, ventilation, and immunisation procedures according to the company’s management regulations, and fed a full-value mixed diet twice daily.

### 2.2. Data Collection

The body weight and sizes of lambs were measured within 24 h after birth and at 60 days. Body sizes mainly included body height, body length, chest circumference, chest depth, chest width and shin circumference, which were measured as follows: Body weight: measured 24 h after birth for newborn lambs and 12 h after fasting for weaned lambs; Body height: measure the vertical distance from the highest point of the dorsal scapula to the ground in the natural upright state of the lamb; Body length: measure the straight line distance from the anterior edge of the scapula to the end of the rump in the natural upright state of the lamb; Chest circumference: measure the length of the posterior end of the scapula around the chest in the natural upright state of the lamb; Chest Width: measure the straight line distance from the widest point of the posterior edge of the scapula on both sides of the scapula in the natural upright state of the lamb; Chest depth: measure the distance from the highest point of the scapula to the base of the sternum in the natural upright state of the lamb; Shin circumference: measure the length around the upper third of the shin of the left forelimb.

### 2.3. FecB Genotyping

Genotyping was performed by Beijing Microread Genetics Co., Ltd. (Beijing, China) using capillary electrophoresis for SNP typing. The principle of capillary electrophoresis is to use a Genetic analyser(ABI) to detect fluorescently labelled DNA fragments, combined with a molecular weight internal standard to calculate the length of the DNA fragments for SNP typing. This method has the advantages of high sensitivity, high accuracy, high throughput, etc. The main reagents and instruments used were as follows: 2 × Buffer B (Beijing Microread Genetics Co., Ltd.); 2G FAST hot initiator (KAPA gene); ROX-500 molecular weight internal standard (Beijing Microread Genetics Co., Ltd.); BC- subMIDI electrophoresis instrument (Beijing Sixty-First Instrument Factory); JY300C electrophoresis tank (Beijing Junyi Oriental Electrophoresis Equipment Co., Ltd., Beijing, China); BioSensSC810B Gel Imager (Shanghai Shanfu Scientific Instrument Co., Ltd., Shanghai, China); NAS-99 Spectrophotometer (ACTGene); GeneAmp9700PCR Instrument (ABI); 3730XLDNAanalyzer (ABI).

The specific method is as follows: after amplifying the target fragment with genomic DNA from blood samples, 1 µL PCR product with 9 µL molecular weight internal standard and formamide mixture (0.5:8.5) was added into a 96-well plate, denatured at 95 °C for 3 min, and then detected on the machine for typing (Table 1).

### 2.4. Statistical Analysis

Allele frequencies and genotype frequencies were calculated for FecB, and the polymorphic information content (PIC), heterozygosity (He), and number of effective alleles (Ne) were calculated using the following equations in Excel.

$$PIC=1-\sum_{i=1}^{1}\;P_{i}^{2}-\sum_{i=1}^{n-1}\sum_{j=i+1}^{n}2P_{i}^{2}\;P_{j}^{2}$$

$$He=1-\sum_{i=1}^{n}P_{i}^{2}$$

$$Ne=\frac{1}{\sum_{i=1}^{n}P_{i}^{2}\;}$$

In the above equation, *n* represents the number of alleles, *P_i_* represents the frequency of the *i*th allele, and *P_j_* is the frequency of the *j*th allele.

Factors involved in this study that influence litter size in ewes are FecB genotype, litter size, and ewe pedigree. The association analysis between FecB and lambing number was carried out by fitting a linear model using SPSS 20.0 software (SPSS, Inc., Chicago, IL, USA) and the model was as follows:

y = μ + G + P + L + e

where y is the observed value, μ is the population mean, G is the FecB genotype effect, P is the litter size effect, L is the pedigree effect, and e is the random error effect.

The SPSS 20.0 software package was used to fit a linear model for meta-analysis of association between FecB and growth traits and the model was as follows:

y = μ + G + e

where y is the phenotypic value, μ is the population mean, G is the FecB genotype effect, and e is the random error effect. Duncan’s method was used for multiple comparisons when differences were significant, and Chi-square test was tested for compliance with Hardy–Weinberg equilibrium of the FecB mutation locus in the experimental population. Chi-square test was used to analyse whether the inheritance patterns of FecB between parents and offspring conformed to Mendel’s Laws of Inheritance.

## 3. Results

### 3.1. Genotyping Results

**Figure 1:**
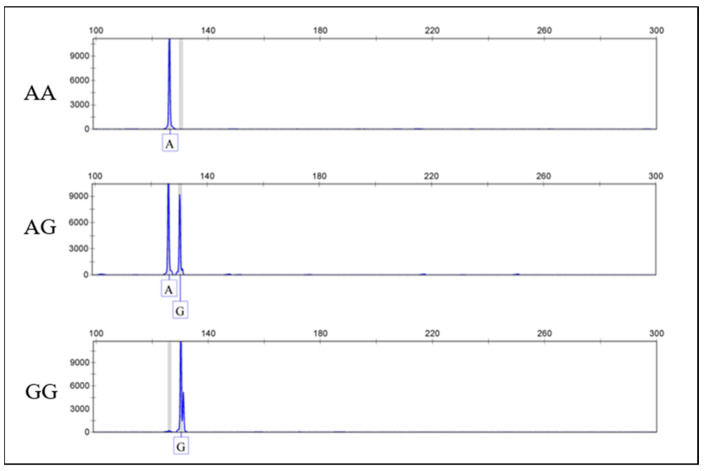
Genotyping results of FecB.

Based on the genotyping results of FecB (Figure 1), it can be obtained that there are three genotypes of FecB in the Suhu meat sheep breeding population, namely the AA genotype, AG genotype, and GG genotype. A population genetic analysis (Table 2) revealed that the dominant genotype in this population was the AG genotype, and the locus was found to be moderately polymorphic (0.25 < *PIC* < 0.5) by analysis. A Chi-square test showed that FecB was in Hardy–Weinberg equilibrium in the early stage of the population (*p* > 0.05), and gradually changed to Hardy–Weinberg disequilibrium (*p* < 0.05) with the selection process. Artificial selection to retain the dominant genotypes and eliminate individuals with the AA genotype from the breeding population during the breeding process was considered.

### 3.2. Effect of FecB on Litter Size and Lamb Survival Rate in the Suhu Meat Sheep Breeding Population

The effect of genotypes of Suhu meat sheep ewes on litter size and lamb survival rate is shown in Table 3. The litter size with the GG genotype of Suhu meat sheep ewes was highly significantly (*p* < 0.01) higher than the Suhu meat sheep ewes with AA and AG genotype, with 1.26 and 0.44 more lambs per litter, respectively; the litter size with the AG genotype of Suhu meat sheep ewes was highly significantly higher than the Suhu meat sheep ewes with the AA genotype (*p* < 0.01), with an average of 0.82 more ewes per litter. Therefore, the effect of FecB on litter size of ewes was GG genotype > AG genotype > AA genotype. Statistical analyses of lamb survival rate revealed that the lamb survival rate with the GG genotype of ewes was highly significantly lower than those with the AA genotype of ewes (*p* < 0.01).

### 3.3. Analysis of Inheritance Patterns of FecB between Parents and Offspring in the Suhu Meat Sheep Breeding Population

The inheritance patterns of FecB between parents and offspring is shown in Table 4. Only the AA genotype exists in the offspring when the mating type is AA × AA. When the mating type was AA × AG, there were two genotypes of AA and AG in the offspring. The actual ratio was 1:1.65, which was consistent with the theoretical ratio of 1:1 (*p* > 0.05). Only the AG genotype exists in the offspring when the mating type is AA × GG. There were three genotypes, AA, AG, and GG, in the offspring with a ratio of 1:2.14:0.74 when the mating type was AG × AG, which was consistent with the theoretical ratio of 1:2:1 (*p* > 0.05). There were two genotypes AG and GG in the offspring with a ratio of 1:0.86 when the mating type was AG × GG, which was consistent with the theoretical ratio of 1:1 (*p* > 0.05). Only the GG genotype exists in the offspring when the mating type is GG × GG.

### 3.4. Effect of FecB on Newborn and Weaning (2 Months of Age) Body Weight and Body Measurements of Suhu Meat Sheep Breeding Population.

In newborn rams, the body weight, body height, body length, and chest circumference in AG genotyped individuals were highly significantly (*p* < 0.01) lower than those in AA genotyped individuals. The chest depth and shin circumference in AG genotyped individuals were significantly (*p* < 0.05) lower than those in AA genotyped individuals, and the body weight, body height, body length, chest circumference, chest depth, chest width, and shin circumference in GG genotyped individuals were highly significantly (*p* < 0.01) lower than those in AA genotyped individuals. In weaned rams, the body weight, chest circumference, and shin circumference in AG genotyped individuals were highly significantly (*p* < 0.01) lower than those in AA genotyped individuals, and the body height in AG genotyped individuals was significantly (*p* < 0.05) lower than those in AA genotyped individuals. The body weight, body height, body length, chest circumference, and shin circumference in GG genotyped individuals were significantly lower than those in AA genotyped individuals (*p* < 0.01), and the chest width in GG genotyped individuals was significantly lower than those in AA genotyped individuals (*p* < 0.05). In weaned ewes, AG genotyped individuals had a highly significantly (*p* < 0.01) lower body weight than AA genotyped individuals, and AG genotyped individuals had a significantly (*p* < 0.05) lower chest circumference than AA genotyped individuals. The body weight, body length, chest circumference, chest depth, and shin circumference in GG genotyped individuals were highly significantly (*p* < 0.01) lower than those in AA genotyped individuals, and the chest circumference in GG genotyped individuals was significantly (*p* < 0.05) lower than those in AG genotyped individuals (Table 5 and Table 6).

## 4. Discussion

As a primary effector gene for improving fertility in ewes, FecB has received widespread attention for improving fertility in ewes by increasing follicle size [5], increasing the number of ovulations [13], influencing reproductive hormone levels in ewes [14], and controlling the expression of reproduction-related genes [15]. In recent years, FecB has been increasingly used for molecular marker-assisted selection breeding, where high-yielding sheep are selected for crossbreeding with low-producing sheep and FecB is introduced into low-producing breeds to improve productivity. Xuewen Ji et al. [16] found that the litter size of ewes in the F1 generation of the AG genotype of Ujimqin and Dorper × Ujimqin sheep was significantly higher than that of ewes of the AA genotype (*p* < 0.05). Yuqing Chong et al. [17] found that the effect of FecB on the litter size of domestic sheep was essentially the same, with each additional copy of the B gene increasing the litter size by 0.4–0.5. Morteza Mahdavi et al. [18] studied Iranian Kalehkoohi sheep and found that BB and B+ genotyped ewes had a highly significantly (*p* < 0.01) higher litter size than ++ genotyped ewes and there was no significant difference in the litter size between BB and B+ genotyped ewes. Chu et al. [19] found that in Small-tail Han sheep population, BB genotyped ewes had 1.4 more litter sizes than ++ genotyped ewes (*p* < 0.01), B+ genotyped ewes had 1.11 more litter sizes than ++ genotyped ewes (*p* < 0.01). In this experiment, the litter size with the GG genotype of ewes was highly significantly higher than that with the AG and AA genotype of ewes, which was basically consistent with the results of the above studies. Gootwine et al. [20] found that in Awassi sheep, the average lamb survival rate of ++ genotyped ewes was 0.98 at birth, while the average lamb survival rate of B+ and BB genotyped ewes decreased to 0.93 and 0.86 at birth. In Assaf sheep, the average lamb survival rate of ++ genotyped ewes was 0.94 at birth, while the average lamb survival rate of B+ and BB genotyped ewes decreased to 0.85 and 0.78 at birth. Sodiq A et al. [21] found that the lamb survival rate of single births (93.62%) was significantly higher than the lamb survival rate of twin births (84.11%) in a Batur sheep population. The results of these studies are in general agreement with the results of the present study. The FecB mutation reduces lamb survival rate, and it is considered to be caused by a higher litter size of ewes with GG genotype and weak newborn lambs. At present, there are few studies on the inheritance patterns of FecB between parents and offspring within a population at domestic and abroad. In this study, the inheritance of FecB follows Mendel’s Laws of Inheritance in all mating types.

FecB plays an important role in the ovary. It can regulate the secretion of FSH and LH which directly affects follicular development and ovulation, and determines litter size in ewes. It has been found that a large number of sinusoidal follicles in ewes carrying FecB mutations (BB, B+) mature prematurely, causing an increase in the number of ovulations. Moreover, the oocytes discharged were smaller in diameter and had significantly fewer GCs in the follicles [22]. In addition, FecB affects the expression of reproduction-related genes. It was found that the expression of the LHCGR gene in the GCs of follicles of ewes carrying the FecB mutation was greater and the expression of the gene was advanced, affecting the proliferation of granulosa cells in the follicle [23].

In addition, FecB has an effect on the growth and development of lambs. E. Gootwine et al. [24] found that the birth weight of lambs with BB genotype and body weight of ewes with BB genotype were significantly lower (*p* < 0.05) than those sheep with ++ and B+ genotype. They had slow growth rates from birth to 5 months of age in Assaf sheep population. Endang Tri Margawati et al. [25] found that the birth weight, weekly weight, and post-weaning daily weight gain of sheep with B+ genotype were higher than those sheep with ++ genotype in MEGA (Merino × Garut) sheep population. All of the above studies have shown that FecB has a detrimental effect on the growth and development of lambs, but there are also relevant studies that have reported a positive effect of FecB on the growth and development of lambs. Feng G et al. [26] found that the body weight, chest circumference, and chest width of sheep with BB and B+ genotype were significantly higher than those sheep with ++ genotype at 90 days in Chinese Merino sheep meat multiparous lines. The results of the present study showed that FecB adversely affects the growth and development of lambs, which is in general agreement with the findings of E. Gootwine et al. and Endang Tri Margawati et al. The results of FecB on lamb growth and development are not uniform, probably due to there being many factors affecting the growth and development of lambs, such as lambing season, litter size, nutritional status of ewes during gestation, and feeding management of lambs, etc., which lead to differences in the effect of FecB on the growth and development of lambs.

## 5. Conclusions

In conclusion, FecB can highly significantly increase the litter size of Suhu meat sheep. However, it will have some adverse effects on the early growth and development. The inheritance of FecB conforms Mendel’s Laws of Inheritance in the Suhu meat sheep breeding population. Therefore, the results of the FecB test and the optimisation of feeding management measures should be applied to improve the reproductive performance of ewes, lamb survival rate and production performance, and thus improve the economic efficiency of sheep farms.

## Figures and Tables

**Table 1:** Reaction system and procedures.

Reagent	10 μL System	Temperature	Time	Cycle
2 × Buffer B	5 µL	95 ℃	5 min	
Primer Mix	2 µL	95 ℃	30 s	35 cycles
2G Fast Taq	0.2 µL	56 ℃	30 s	
DNA	1.0 µL	72 ℃	30 s	
ddH_2_O	to 10 µL	60 ℃	30 min	

**Table 2:** Population genetics analysis of the FecB locus in Suhu meat sheep breeding population.

Time	Genotype Frequency			Allele Frequency		PIC	He	Ne	*p*-Value
	AA	AG	GG	A	G				
2022/1/21	0.25 (97)	0.50 (190)	0.25 (95)	0.503	0.497	0.375	0.500	2.000	0.99
2022/11/10	0.21 (173)	0.52 (429)	0.27 (223)	0.470	0.530	0.374	0.498	1.993	0.45
2023/2/26	0.12 (122)	0.55 (567)	0.33 (347)	0.391	0.609	0.363	0.476	1.910	0.00

*p* < 0.05 indicates in Hardy–Weinberg disequilibrium, with sample size in parentheses.

**Table 3:** Effect of FecB on litter size of Suhu meat sheep breeding population.

Genotype	Litter Size			Lamb Survival Rate/%
	Primiparous Ewes	Parturient Ewes	Average Litter Size	
AA	/	1.14 ± 0.45 (28)	1.14 ± 0.45 ^C^ (28)	100 ^Aa^ (19/19)
AG	1.98 ± 0.72 (42)	1.96 ± 0.67 (170)	1.96 ± 0.68 ^B^ (212)	94.6 ^Ab^ (334/356)
GG	2.41 ± 1 (150)	2.37 ± 0.81 (83)	2.40 ± 0.93 ^A^ (233)	90.8 ^B^ (436/480)

Data in the same column are labelled with different capital letters on the shoulder to represent highly significant differences (*p* < 0.01), and lowercase letters to represent significant differences (*p* < 0.05). Sample sizes are shown in parentheses, and results are presented as the mean ± standard deviation.

**Table 4:** Inheritance patterns of FecB between parents and offspring in the Suhu meat sheep breeding population.

Mating Type	Number of Offspring	Genotype	Quantitative Ratio	Actual Ratio	Theoretical Ratio	*p*-Value
AA × AA	4	AA	/	/	/	/
AA × AG	45	AA:AG	17:28	1:1.65	1:1	0.101
AA × GG	123	AG	/	/	/	/
AG × AG	136	AA:AG:GG	35:75:26	1:2.14:0.74	1:2:1	0.268
AG × GG	271	AG:GG	157:135	1:0.86	1:1	0.198
GG × GG	64	GG	/	/	/	/

*p*-values were obtained by Chi-square Test.

**Table 5:** Effect of FecB on body size and weight of newborn lambs of Suhu meat sheep.

Sex	Genotype	Body Weight	Body Height	Body Length	Chest Circumference	Chest Depth	Chest Width	Shin Circumference
Male (312)	AA	4.26 ± 0.14 ^A^	39.31 ± 0.5 ^A^	31.49 ± 0.45 ^A^	36.04 ± 0.5 ^A^	15.32 ± 0.27 ^Aa^	10.55 ± 0.26 ^A^	6.25 ± 0.13 ^Aa^
	AG	3.55 ± 0.07 ^B^	37.58 ± 0.25 ^B^	30.07 ± 0.22 ^B^	34.41 ± 0.25 ^B^	14.57 ± 0.13 ^Ab^	10.14 ± 0.13 ^A^	5.96 ± 0.07 ^Ab^
	GG	3.44 ± 0.09 ^B^	36.81 ± 0.32 ^B^	28.62 ± 0.29 ^C^	33.17 ± 0.32 ^C^	13.7 ± 0.17 ^B^	9.23 ± 0.17 ^B^	5.59 ± 0.08 ^B^
Female (569)	AA	4.14 ± 0.08 ^A^	38.39 ± 0.39 ^Aa^	31.57 ± 0.29 ^A^	36.66 ± 1.31	15.83 ± 0.22 ^A^	10.2 ± 0.16 ^A^	5.98 ± 0.26
	AG	3.63 ± 0.04 ^B^	37.31 ± 0.22 ^b^	30.59 ± 0.16 ^B^	35.93 ± 0.74	15.08 ± 0.12 ^B^	10.23 ± 0.09 ^A^	6.04 ± 0.15 ^a^
	GG	3.25 ± 0.06 ^C^	36.99 ± 0.31 ^B^	29 ± 0.23 ^C^	33.41 ± 1.06	14.06 ± 0.18 ^C^	9.41 ± 0.13 ^B^	5.43 ± 0.21 ^b^

Data in the same column are labelled with different capital letters on the shoulder to represent highly significant differences (*p* < 0.01), and lowercase letters to represent significant differences (*p* < 0.05). Sample sizes are shown in parentheses, and results are presented as the mean ± standard deviation.

**Table 6:** Effect of FecB on body size and weight of weaned lambs of Suhu meat sheep.

Sex	Genotype	Body Weight	Body Height	Body Length	Chest Circumference	Chest Depth	Chest Width	Shin Circumference
Male (312)	AA	20.87 ± 0.7 ^A^	52.66 ± 0.54 ^Aa^	55.12 ± 0.76 ^A^	61.82 ± 0.83 ^A^	24.29 ± 0.41	17.61 ± 0.4 ^a^	7.5 ± 0.13 ^A^
	AG	18.11 ± 0.35 ^B^	51.21 ± 0.27 ^b^	53.21 ± 0.38	58.58 ± 0.42 ^B^	23.77 ± 0.2	17.05 ± 0.2	7.12 ± 0.06 ^B^
	GG	17.81 ± 0.45 ^B^	50.88 ± 0.35 ^B^	52 ± 0.49 ^B^	57.89 ± 0.53 ^B^	23.54 ± 0.26	16.53 ± 0.26 ^b^	7 ± 0.08 ^B^
Female (569)	AA	19.11 ± 0.41 ^A^	49.74 ± 0.37	52.43 ± 0.48 ^A^	60.98 ± 0.56 ^Aa^	23.15 ± 0.9 ^A^	16.55 ± 0.25	7.02 ± 0.07 ^A^
	AG	17.85 ± 0.23 ^B^	50.07 ± 0.21	52.74 ± 0.27 ^A^	59.56 ± 0.31 ^Ab^	24.13 ± 0.5 ^a^	16.9 ± 0.14	6.92 ± 0.04 ^A^
	GG	16.31 ± 0.34 ^C^	49.57 ± 0.3	50.75 ± 0.39 ^B^	57.43 ± 0.45 ^B^	22.91 ± 0.73 ^Bb^	15.79 ± 0.2	6.55 ± 0.05 ^B^

Data in the same column are labelled with different capital letters on the shoulder to represent highly significant differences (*p* < 0.01), and lowercase letters to represent significant differences (*p* < 0.05). Sample sizes are shown in parentheses, and results are presented as the mean ± standard deviation.

## Data Availability

Data are contained within the article.
